# Advanced Monocular Outdoor Pose Estimation in Autonomous Systems: Leveraging Optical Flow, Depth Estimation, and Semantic Segmentation with Dynamic Object Removal

**DOI:** 10.3390/s24248040

**Published:** 2024-12-17

**Authors:** Alireza Ghasemieh, Rasha Kashef

**Affiliations:** Electrical, Computer, and Biomedical Engineering, Toronto Metropolitan University, Toronto, ON M5B 2K3, Canada; alireza.ghasemieh@torontomu.ca

**Keywords:** pose estimation, visual odometry, optical flow, depth estimation, semantic segmentation

## Abstract

Autonomous technologies have revolutionized transportation, military operations, and space exploration, necessitating precise localization in environments where traditional GPS-based systems are unreliable or unavailable. While widespread for outdoor localization, GPS systems face limitations in obstructed environments such as dense urban areas, forests, and indoor spaces. Moreover, GPS reliance introduces vulnerabilities to signal disruptions, which can lead to significant operational failures. Hence, developing alternative localization techniques that do not depend on external signals is essential, showing a critical need for robust, GPS-independent localization solutions adaptable to different applications, ranging from Earth-based autonomous vehicles to robotic missions on Mars. This paper addresses these challenges using Visual odometry (VO) to estimate a camera’s pose by analyzing captured image sequences in GPS-denied areas tailored for autonomous vehicles (AVs), where safety and real-time decision-making are paramount. Extensive research has been dedicated to pose estimation using LiDAR or stereo cameras, which, despite their accuracy, are constrained by weight, cost, and complexity. In contrast, monocular vision is practical and cost-effective, making it a popular choice for drones, cars, and autonomous vehicles. However, robust and reliable monocular pose estimation models remain underexplored. This research aims to fill this gap by developing a novel adaptive framework for outdoor pose estimation and safe navigation using enhanced visual odometry systems with monocular cameras, especially for applications where deploying additional sensors is not feasible due to cost or physical constraints. This framework is designed to be adaptable across different vehicles and platforms, ensuring accurate and reliable pose estimation. We integrate advanced control theory to provide safety guarantees for motion control, ensuring that the AV can react safely to the imminent hazards and unknown trajectories of nearby traffic agents. The focus is on creating an AI-driven model(s) that meets the performance standards of multi-sensor systems while leveraging the inherent advantages of monocular vision. This research uses state-of-the-art machine learning techniques to advance visual odometry’s technical capabilities and ensure its adaptability across different platforms, cameras, and environments. By merging cutting-edge visual odometry techniques with robust control theory, our approach enhances both the safety and performance of AVs in complex traffic situations, directly addressing the challenge of safe and adaptive navigation. Experimental results on the KITTI odometry dataset demonstrate a significant improvement in pose estimation accuracy, offering a cost-effective and robust solution for real-world applications.

## 1. Introduction

Pose estimation is a crucial aspect in robotics, autonomous driving, and computer vision, enabling the accurate determination of an object’s orientation and position in space. While significant research has been dedicated to pose estimation using LiDAR or stereo cameras, there remains a notable gap in the literature concerning monocular vision systems—those utilizing only a single camera. Most existing solutions rely heavily on LiDAR sensors or stereo camera setups, which, although highly accurate, present significant limitations in weight, cost, and complexity. In contrast, with its practicality and cost-effectiveness, monocular vision is a prevalent choice for many drones, cars, and other autonomous vehicles. However, developing robust and reliable pose estimation models that leverage monocular images remains underexplored. The practicality and cost-effectiveness of monocular vision cannot be overstated. Many drones, cars, and other autonomous vehicles are equipped with only one camera due to the simplicity and affordability of such configuration. Despite this, developing robust and reliable pose estimation models that leverage monocular images remains underexplored. The lack of research in this area is evident when reviewing the KITTI benchmark leaderboard, where most high-performing models are based on LiDAR or stereovision datasets, leaving monocular approaches relatively underrepresented. Our work aims to fill this gap, providing a practical and cost-effective solution for the industry.

Our work aims to bridge this research gap by presenting a novel approach for pose estimation using monocular images. This is particularly crucial for applications where deploying additional sensors is not feasible due to cost or physical constraints. By focusing on a single-camera setup, we seek to develop a model that meets the performance standards set by multi-sensor systems and leverages the inherent advantages of monocular vision, such as reduced weight and cost. In this paper, we introduce an advanced framework for monocular visual odometry that integrates state-of-the-art preprocessing techniques, including optical flow, depth estimation, and semantic segmentation. Optical flow provides essential motion information between consecutive frames, depth estimation offers spatial context by predicting distances within the scene, and semantic segmentation enhances the understanding of object boundaries and scene context to remove the negative impact of dynamic objects on pose estimation. Combining these techniques, our approach aims to deliver high accuracy and robustness in pose estimation from a single camera input. This research contributes to the field by addressing the practical need for effective monocular pose estimation models. It highlights the potential of monocular systems to provide reliable performance in real-world applications, paving the way for broader adoption of cost-effective and lightweight vision-based solutions in various autonomous systems. Through extensive experiments on the KITTI odometry dataset, we demonstrate the effectiveness of our proposed framework, underscoring its potential to fill the existing gap in monocular pose estimation research. The main contributions of this paper can be summarized as follows:

1. **Novel Monocular Pose Estimation Framework**: This paper introduces an advanced framework for pose estimation using monocular images, addressing the gap in research where most high-performing models rely on multi-sensor setups like LiDAR or stereo cameras. The proposed approach focuses on monocular vision, which is cost-effective and practical for various autonomous systems.
2. **Integration of Optical Flow, Depth Estimation, and Semantic Segmentation**: The proposed framework combines optical flow, depth estimation, and semantic segmentation to improve the accuracy and robustness of visual odometry. These preprocessing steps allow the system to gather more environmental information, such as motion, spatial depth, and scene context.
3. **Dynamic Object Removal**: An essential contribution is the dynamic object removal technique that filters out moving objects (e.g., cars, pedestrians) from the scene, reducing noise in pose estimation and focusing on static background elements. This improves pose accuracy in dynamic environments.
4. **Cost-effective and Lightweight Solution**: By focusing on monocular vision and minimizing the need for additional sensors, this paper presents a lightweight and adaptable solution, making it highly suitable for applications in resource-constrained environments such as drones and autonomous vehicles.
5. **Experimental Validation**: The framework’s performance is validated through experiments on the KITTI odometry dataset, demonstrating significant improvements in pose estimation accuracy compared to existing methods. The experimental results highlight the superiority of the proposed approach, particularly when combining optical flow, depth estimation, and semantic segmentation.

These contributions collectively advance the field of monocular pose estimation by offering a practical, efficient, and robust solution for autonomous systems operating in GPS-denied and dynamic environments. The rest of this paper is organized as follows: Section 2 provides an overview of related work focusing on preprocessing techniques, including optical flow, depth estimation, and semantic segmentation. Section 3 introduces vision-based pose estimation. Section 4 discusses the methodology used in this research, explaining the proposed pose estimation framework, its architecture, and the integration of optical flow, depth, and semantic segmentation. Section 4 covers the experimental setup, including dataset details, training procedures, and evaluation metrics. The results of the experiments, as well as a comparison of different preprocessing methods, are presented in Section 5. Finally, Section 6 concludes this paper by summarizing our findings and discussing potential future research directions.

## 2. Related Work

This section presents related work pertinent to our preprocessing techniques, focusing on optical flow, depth estimation, and semantic segmentation. Optical flow, a technique used to estimate the motion of objects between consecutive frames, has been extensively studied and applied in various computer vision tasks, particularly in motion tracking and object detection. Depth estimation is a method to estimate the distance of each pixel from the camera. It helps to generate a new data type out of an image for further applications. The advancements in semantic segmentation have enhanced the understanding of scene context and object boundaries, which are crucial for tasks like scene parsing and robotic perception. By integrating these advanced preprocessing techniques, our approach aims to leverage the strengths of each method, leading to improved accuracy and robustness in visual odometry.

### 2.1. Optical Flow

This subsection reviews the most recent proposed optical flow methods. Luo et al. [1] introduce a novel graph-based approach, Adaptive Graph Reasoning for Optical Flow (AGFlow), which aims to enhance optical flow estimation by incorporating scene/context information. By decoupling context reasoning from the matching procedure and leveraging adaptive graph reasoning, AGFlow outperforms existing methods in accuracy. The results demonstrate that AGFlow achieves the best accuracy on Sintel clean and final passes, with an Endpoint Error (EPE) of 1.43 and 2.47 pixels, surpassing state-of-the-art approaches by 11.2% and 13.6%, respectively. Their work highlights the significance of context information in improving motion estimation in video analysis. Jeong et al. [2] proposed a method for improving optical flow estimation by imposing consistency on distrained pairs. Their study focused on enhancing the accuracy of optical flow estimation in computer vision applications. By addressing distrained pairs, the researchers significantly improved the precision and robustness of optical flow estimation algorithms. Bai et al. [3] proposed a novel method for optical flow estimation using deep equilibrium models. Their study focused on leveraging deep learning techniques to achieve accurate optical flow estimation by formulating the problem and finding the equilibrium of a dynamic system. Through extensive experiments, the authors demonstrated that their approach outperformed existing methods in accuracy and robustness, showcasing the potential of deep equilibrium models in computer vision. Huang et al. [4] introduce FlowFormer, a transformer-based network architecture for optical flow estimation, enhanced by the Masked Cost Volume AutoEncoding (MCVA) pretraining method. FlowFormer tokenizes the cost volume from image pairs and refines flow estimation iteratively using a cost volume encoder-decoder architecture. Their study demonstrates that FlowFormer achieves notable performance improvements, with FlowFormer + MCVA ranking 1st on Sintel and KITTI-2015 benchmarks, achieving 7.76% and 7.18% error reductions from FlowFormer on the Sintel benchmark. Incorporating MCVA, which leverages unlabeled data for pretraining, they showcase the potential for enhancing optical flow estimation models without needing costly ground truth data. Wan et al. [5] introduced an innovative deep-learning approach for estimating dense and continuous optical flow using event camera data from a single image. By leveraging this framework, their research enhances the precision in detecting rapid movements, contributing significantly to computer vision and advancing optical flow estimation techniques. Deng et al. [6] presented a novel approach for optical flow estimation by explicitly disentangling motion components, leading to enhanced efficiency. Their method showcases superior performance in accurately estimating optical flow by separating complex motion patterns into distinct components, thereby improving computational efficiency without compromising accuracy. Their study demonstrates significant advancements in optical flow estimation through rigorous experimentation, making it a promising technique for various computer vision applications. Luo et al. [7] proposed a novel approach for learning optical flow using Kernel Patch Attention. Their method leverages attention mechanisms to focus on informative image regions, enhancing the accuracy of optical flow estimation. Experimental results demonstrate that the proposed technique outperforms existing methods in terms of accuracy and computational efficiency, showcasing its potential for advancing optical flow estimation in computer vision applications. Zhao et al. [8] propose a novel approach for optical flow estimation titled “Global Matching with Overlapping Attention”. Their method leverages overlapping attention mechanisms to enhance the accuracy of optical flow estimation. Through extensive experiments, the authors demonstrate that their approach outperforms existing methods in accuracy and robustness, making it a promising advancement in computer vision. In their recent work, Hu et al. [9] delve into optical flow estimation specifically tailored for spiking cameras. The study focuses on developing algorithms that can accurately estimate optical flow using data captured by spiking cameras, a novel approach in computer vision. Their results showcase promising advancements in optical flow estimation techniques, particularly in scenarios where traditional cameras may not be as effective, opening up new possibilities for applications in robotics, autonomous vehicles, and surveillance systems. Tu et al. [10] provide a comprehensive survey on the use of optical flow for video super-resolution, focusing on applications in computer vision and motion compensation. Their study delves into techniques and algorithms that enhance video quality through optical flow methods. Results from their survey highlight the effectiveness of optical flow in improving video resolution and motion estimation, offering valuable insights for researchers and practitioners in the field. Guizilini et al. [11] propose a novel approach for learning optical flow, depth, and scene flow without needing real-world labels. The model can accurately estimate these crucial visual perception tasks by leveraging unsupervised learning techniques. Their results showcase the effectiveness of the proposed method in achieving state-of-the-art performance without the reliance on labeled data, offering a promising avenue for advancing computer vision applications. Liang et al. [12] introduce a novel approach for learning optical flow using multiplane images. Their study demonstrates that the model can generate more realistic optical flow predictions by leveraging multiplane images compared to traditional methods. Their results show significant improvements in optical flow accuracy and visual quality, highlighting the effectiveness of incorporating multiplane images in optical flow estimation tasks. Han et al. [13] present RealFlow, a method for generating realistic optical flow datasets from videos using an Expectation-Maximization (EM) approach. Their technique aims to enhance the quality and authenticity of optical flow datasets, which is crucial for computer vision tasks. By employing EM-based generation, their study demonstrates improved accuracy and realism in optical flow datasets, which can benefit various applications in computer science, particularly in developing and evaluating algorithms for motion analysis and object tracking. Luo [14] introduces GAFlow, a novel approach integrating Gaussian attention mechanisms into optical flow estimation. Their method enhances the optical flow estimation process by leveraging Gaussian attention to focus on relevant image regions, improving the accuracy of flow predictions. Experimental results demonstrate that GAFlow outperforms existing optical flow methods in accuracy and robustness, showcasing its potential for advancing optical flow estimation in computer vision applications. Xu et al. [15] proposed GMFlow, a novel approach for learning optical flow through global matching. Their method achieves state-of-the-art results in optical flow estimation tasks by leveraging the softmax function. Their study, published in 2022, significantly advances computer science by providing a more effective and efficient solution for optical flow computation. Shi et al. [16] focused on enhancing optical flow estimation by leveraging temporal information from multiple frames. The proposed VideoFlow method demonstrates significant improvements in accuracy and robustness compared to existing techniques, showcasing the effectiveness of incorporating temporal cues in optical flow estimation tasks. By exploiting the temporal coherence between frames, VideoFlow achieves state-of-the-art performance, making it a promising approach for various computer vision applications requiring precise motion estimation. The authors introduced a novel pretraining optical flow estimation model approach using Masked Cost Volume Autoencoding. This method aims to enhance the accuracy and robustness of optical flow estimation by leveraging masked cost volumes during the pretraining phase. Their results demonstrate significant improvements in optical flow estimation performance compared to traditional methods, showcasing the effectiveness of the proposed FlowFormer++ approach in advancing the field of optical flow estimation. Dong, Cao, and Fu [17] present a novel approach to optical flow estimation by reevaluating it through the lens of geometric matching consistent perspective. Their paper introduces a framework that leverages geometric constraints to improve the accuracy and robustness of optical flow estimation. Through extensive experiments on benchmark datasets, the proposed method demonstrates superior performance compared to existing optical flow techniques, showcasing its potential for advancing the field of computer vision. Zheng et al. [18] propose a novel approach named Deep Inverse Patchmatch (DIP) for enhancing high-resolution optical flow. DIP aims to improve the accuracy and generalization capabilities of optical flow estimation by leveraging deep learning techniques. Their results demonstrate that DIP outperforms existing methods in terms of accuracy and efficiency, showcasing its potential for advancing the field of computer vision and optical flow analysis. Their paper introduces CRAFT [19], a Cross-Attentional Flow Transformer designed to enhance the robustness of optical flow estimation. CRAFT effectively captures long-range dependencies in image sequences using cross-attention mechanisms, improving optical flow accuracy. Experimental results demonstrate that CRAFT outperforms existing methods in challenging scenarios, showcasing its potential for advancing optical flow estimation in computer vision applications.

### 2.2. Depth Estimation

This subsection reviews the most recent proposed depth estimation methods. Lyu et al. [20] focused on enhancing monocular depth estimation through self-supervised learning. The proposed HR-Depth model addresses the challenge of inaccurate depth estimation in large gradient regions by improving high-resolution features with spatial and semantic information. This is achieved by redesigning skip connections in DepthNet and incorporating a feature fusion Squeeze-and-Excitation (fSE) module. The network architecture utilizes Resnet-18 as the encoder. It outperforms previous state-of-the-art methods in terms of accuracy with fewer parameters, surpassing complex models like Monodepth2 using a lightweight network based on MobileNetV3. Experimental results demonstrate that the lightweight network achieves comparable performance to larger models while being more efficient, with only 20% of the parameters. Their findings highlight the significance of leveraging high-resolution features and efficient feature fusion techniques in improving monocular depth estimation accuracy. By introducing innovative strategies in network design, such as the fSE module and optimized skip connections, the HR-Depth model demonstrates superior performance compared to existing state-of-the-art methods. The emphasis on achieving high accuracy with reduced parameters through a lightweight network like MobileNetV3 showcases the practical applicability of the proposed approach in real-world scenarios, making it a promising advancement in computer vision for depth estimation. Yinhao et al. [21] focused on enhancing depth estimation accuracy for multi-view 3D object detection through the BEVDepth method. The main idea revolves around utilizing explicit depth supervision to improve the reliability of depth acquisition. The network architecture likely involves incorporating depth supervision mechanisms within the existing multi-view object detection framework to enhance the precision of depth estimation. Numerical results are expected to demonstrate the superior performance of BEVDepth in accurately determining object depths in a multi-view setup, showcasing advancements in 3D object detection accuracy. By leveraging explicit depth supervision, the BEVDepth method addresses the challenges of acquiring reliable depth information for multi-view 3D object detection. The network architecture likely integrates depth supervision techniques into the existing detection framework to enhance the robustness and accuracy of depth estimation. The numerical results are anticipated to showcase the effectiveness of BEVDepth in improving the precision of depth estimation, thereby advancing the capabilities of multi-view 3D object detection systems. Li et al. [22] discuss the integration of Transformer and convolutional neural networks for monocular depth estimation. Their paper highlights the strengths of the Transformer in capturing long-range correlations and the effectiveness of convolution in handling local information. Combining these two approaches, the proposed DepthFormer architecture aims to achieve accurate depth estimation by leveraging long-range dependencies and regional details. The network architecture of DepthFormer likely involves a hybrid design that incorporates Transformer layers to capture global context and convolutional layers to extract local features. This combination allows the model to effectively fuse information from distant and nearby regions in the input image, enhancing the accuracy of monocular depth estimation. The numerical results presented in their paper are expected to demonstrate the superior performance of DepthFormer compared to existing methods, showcasing its ability to leverage both long-range correlation and local information for more precise depth estimation tasks. Bhat et al. [23] introduced AdaBins, a novel approach for depth estimation leveraging adaptive bins. The main idea revolves around utilizing a set of adaptive bins to estimate depth maps efficiently. Their method involves a hierarchical architecture where each bin estimates a specific depth range, enabling the network to capture fine-grained details in the depth estimation process. The network architecture comprises multiple layers of adaptive bins, each refining the depth estimation progressively. Through extensive experimentation, the authors demonstrate the effectiveness of AdaBins in accurately estimating depth maps, outperforming existing methods in terms of accuracy and computational efficiency. In their work, Bhat et al. present numerical results showcasing the superior performance of AdaBins in-depth estimation tasks. The network architecture’s adaptability to different depth ranges is highlighted through quantitative evaluations, demonstrating improved accuracy compared to traditional methods. Their results indicate that AdaBins achieves state-of-the-art performance in depth estimation and shows robustness in handling complex scenes and varying lighting conditions. Overall, numerical findings underscore the efficacy of AdaBins in advancing the field of depth estimation through its innovative use of adaptive bins. Hoyer et al. [24] proposed a novel approach to enhance semi-supervised and domain-adaptive semantic segmentation through self-supervised depth estimation. The authors introduce a robust data augmentation technique combining images and labels based on the scene’s geometry. By leveraging this method, the network can learn from limited labeled data and adapt to diverse domains, improving segmentation accuracy across various scenarios. The incorporation of solid data augmentation by merging images and labels using scene geometry, as outlined, demonstrates a significant advancement in enhancing semi-supervised and domain-adaptive semantic segmentation. This approach enables the network to learn more effectively from limited labeled data. It enhances its adaptability to different domains, improving segmentation performance in various real-world applications. The proposed method by Ramamonjisoa et al. [25] introduces a novel approach for monocular depth prediction using wavelet decomposition. Their method leverages a fully differentiable encoder-decoder framework integrated with wavelet decomposition to predict depth maps efficiently. Their study demonstrates the ability to learn wavelet coefficients without direct supervision, achieving high-fidelity depth reconstructions through the inverse wavelet transform. Furthermore, their research showcases the feasibility of self-supervised learning for wavelet coefficients, even without ground truth depth information. When applied to various monocular depth estimation models, the proposed method consistently outperforms or matches the original models while reducing computational complexity in the decoder network by over 50%. The architecture employed in the experiments is a modified version of the model utilized in a previous study, as outlined in the primary publication. The research achieves efficient depth prediction from monocular images by integrating wavelet decomposition into the encoder-decoder structure. This innovative approach not only enhances the accuracy of depth maps but also demonstrates the potential for learning wavelet coefficients autonomously, showcasing promising results in self-supervised scenarios. Their findings underscore the efficacy of wavelet decomposition in improving monocular depth estimation models, offering a more efficient alternative with comparable or superior performance to existing methods. Yuan et al. [26] introduced a novel approach for monocular depth estimation using Neural Window Fully Connected Conditional Random Fields (CRFs). The main idea revolves around leveraging CRFs to refine depth predictions by incorporating contextual information from neighboring pixels. The method involves constructing a fully connected CRF graph where each node represents a pixel and utilizing neural networks to learn the pairwise potentials for the CRF. By integrating the CRF into the depth estimation pipeline, the model can effectively capture long-range dependencies and enhance the spatial coherence of depth maps. Regarding network architecture, the proposed method integrates the CRF module into the existing depth estimation network. The CRF module consists of a fully connected graph where each node corresponds to a pixel in the input image. The pairwise potentials in the CRF are learned using neural networks, allowing the model to capture complex relationships between pixels. During inference, the CRF refines the initial depth predictions by considering the global context of the image, leading to more accurate depth estimations. Numerical results presented in the study demonstrate the effectiveness of the proposed Neural Window Fully connected CRFs for monocular depth estimation. The method outperforms existing approaches regarding depth accuracy and spatial consistency, showcasing the benefits of incorporating CRFs into the depth estimation pipeline. The experimental results highlight the potential of the proposed approach to improve the quality of depth predictions in computer vision tasks. Li et al. [27] delved into stereo-depth estimation through a novel sequence-to-sequence approach leveraging transformers. The main idea revolves around enhancing depth estimation accuracy by incorporating transformer networks to process stereo image sequences. The method involves encoding stereo image pairs into a sequence and utilizing transformer layers to predict depth maps, demonstrating improved performance compared to traditional stereo matching algorithms. The network architecture comprises an encoder-decoder structure with transformer layers, enabling the model to capture long-range dependencies in stereo image sequences. The encoder processes the input stereo images, while the decoder generates depth maps through transformer-based sequence-to-sequence modeling. The transformer architecture facilitates effective information propagation across the sequence, enhancing the model’s ability to estimate accurate depth information. Numerical results showcase superior depth estimation accuracy and robustness compared to conventional stereo matching techniques, highlighting the efficacy of the proposed transformer-based approach in stereo depth estimation tasks. Zhao et al. [28] suggested MonoViT, a novel approach for self-supervised monocular depth estimation utilizing a Vision Transformer (ViT). The main idea revolves around leveraging the ViT architecture to learn depth from a single image without requiring stereo pairs. The method involves pretraining the ViT on a large-scale dataset using a self-supervised framework that predicts depth maps from individual images. The network architecture of MonoViT consists of a ViT backbone that processes the input image patches and a depth decoder that generates the depth map. The ViT is adapted to the depth estimation task by incorporating positional encodings and modifying the self-attention mechanism. The depth decoder refines the depth predictions through a multiscale architecture that fuses features from different levels of the network. Experimental results demonstrate that MonoViT achieves state-of-the-art performance on various benchmark datasets, surpassing previous methods in accuracy and generalization to diverse scenes. Chen et al. [29] provided an attention based context aggregation network for monocular depth estimation. The main idea revolves around leveraging attention mechanisms to aggregate contextual information effectively for depth estimation tasks. The network architecture incorporates attention modules to selectively focus on relevant features, enhancing the model’s ability to capture intricate spatial dependencies within the input data. The authors demonstrate the network’s efficacy in improving depth estimation accuracy through extensive experimentation, showcasing superior performance compared to existing methods. Numerical results indicate significant advancements in prediction accuracy, highlighting the effectiveness of the proposed attention-based approach. Chen et al. delve into the intricate details of the attention-based context aggregation network for monocular depth estimation in their research. The methodology involves integrating attention mechanisms within the network architecture to facilitate the aggregation of contextual information from the input data. The model can effectively capture long-range dependencies and intricate spatial relationships by strategically incorporating attention modules, enhancing depth estimation performance. The numerical results presented in the study showcase substantial improvements in depth prediction accuracy, underscoring the efficacy of the proposed approach in advancing state-of-the-art monocular depth estimation within the realm of computer vision. Miangoleh et al. [30] focused on enhancing monocular depth estimation models to achieve high-resolution outputs through a content adaptive multi-resolution merging approach. The main idea involves leveraging a multi-resolution strategy that adapts to the content of the input image to improve the accuracy and quality of depth estimation. The method integrates information from multiple resolutions in a content-aware manner, enhancing the model’s ability to capture intricate details in the depth map. The network architecture in this research comprises a multi-resolution fusion module that dynamically combines features from different scales to refine the depth estimation. By incorporating content adaptive mechanisms, the model can effectively handle various complexities in the input images, leading to more precise depth predictions. The numerical results demonstrate significant improvements in the estimation accuracy, particularly in challenging scenarios with intricate textures and fine details, showcasing the efficacy of the proposed content adaptive multi-resolution merging technique. Hoyer et al. [31] enhanced semantic segmentation through self-supervised depth estimation. The main idea revolves around leveraging depth information to refine semantic segmentation accuracy. The authors propose three key methods to achieve this: incorporating depth cues into the segmentation network, utilizing depth information for data augmentation, and employing depth estimation as a self-supervised task to enhance feature learning. In terms of network architecture, the authors introduce a novel framework that integrates depth estimation modules into the semantic segmentation network. This architecture allows for the joint learning of depth and semantic segmentation tasks, leading to improved segmentation performance. The numerical results demonstrate significant enhancements in segmentation accuracy when incorporating self-supervised depth estimation, showcasing the effectiveness of the proposed methods in advancing semantic segmentation tasks.

### 2.3. Semantic Segmentation

This subsection reviews the most recent proposed semantic segmentation methods. Wang et al. [32] focus on Deep High-Resolution Representation Learning for Visual Recognition, emphasizing the creation of semantically enriched and spatially precise representations. By leveraging subnetworks within computer science, the approach aims to enhance the quality and accuracy of visual recognition systems through advanced representation learning techniques. Fan et al. [33] introduce a novel approach to real-time semantic segmentation by reevaluating the BiSeNet model. Their research focuses on enhancing the efficiency of semantic segmentation inference, particularly in the context of computer science applications. By revisiting the BiSeNet architecture, their study aims to optimize real-time semantic segmentation performance, offering insights into advanced techniques for efficient image analysis. Huynh et al. [34] introduce a novel approach termed Progressive Semantic Segmentation, which aims to enhance the accuracy and efficiency of pixelwise classification in computer vision tasks. By progressively refining segmentation results through iterative processes, the model achieves an improvement in the semantic understanding of images, contributing to advancements in the field of computer science. The methodology focuses on refining segmentation masks at different resolutions, leading to more precise object delineation and improved performance in semantic segmentation tasks. Xu [35] introduces PIDNet, a real-time semantic segmentation network inspired by PID controllers. This innovative approach leverages the principles of Proportional-Integral-Derivative control to enhance the efficiency and accuracy of semantic segmentation tasks. By incorporating PID control mechanisms into the network architecture, PIDNet aims to optimize real-time semantic segmentation performance by dynamically adjusting segmentation outputs based on error feedback. Yan & Zheng [36] explore the utilization of HRNet and PSPNet for multiband semantic segmentation in remote sensing images. HRNet and PSPNet are employed to enhance the accuracy and efficiency of semantic segmentation tasks in the context of remote sensing imagery. By leveraging these advanced neural computing techniques, their research aims to improve pixel-level classification accuracy. Strudel et al. [37] introduced Segmenter, a transformer-based model tailored for semantic segmentation tasks in computer vision. The model leverages transformer architecture to enhance pixelwise classification accuracy, demonstrating promising results in improving semantic segmentation performance. Segmenter offers a novel approach to semantic segmentation by incorporating transformer mechanisms, showcasing advancements in utilizing transformer models for pixel-level classification in computer vision applications. Yuan et al. [38] introduced OCNet, which leverages object context for semantic segmentation tasks in computer vision. The approach enhances pixel-level classification accuracy by incorporating object-level information into the segmentation process. By integrating object context, OCNet aims to improve the precision and efficiency of semantic segmentation algorithms, particularly in scenarios where object relationships play a crucial role in accurate pixelwise classification. Wu et al. [39] focused on optimizing HRNet for image semantic segmentation, a critical task in computer vision. HRNet, known for its high-resolution representations, is fine-tuned to enhance its performance in accurately segmenting images at the pixel level. By optimizing HRNet, the researchers aim to improve the efficiency and accuracy of semantic segmentation tasks, contributing to advancements in computer vision applications. Xu et al. [40] introduce HRCNet, a CNN architecture designed for the semantic segmentation of remote sensing images (RSIs), addressing challenges such as spatial information loss, global context neglect, and category scale imbalance. HRCNet leverages HRNet to preserve spatial information, incorporates a lightweight dual attention (LDA) module for global context, and employs a Feature Enhancement Feature Pyramid (FEFP) module and a Boundary-Aware (BA) module with a boundary-aware loss function to improve segmentation and boundary detection. Evaluated on Potsdam and Vaihingen datasets, HRCNet achieves significant improvements, demonstrating its effectiveness in enhancing semantic segmentation by integrating high-resolution features, attention mechanisms, and boundary-aware strategies.

The comparative summary in Table 1 highlights the performance of each method, emphasizing how they tackle specific challenges in their respective domains. 

The research gap identified in this paper lies in the limited exploration and development of robust monocular vision-based pose estimation models, particularly for outdoor applications in GPS-denied environments. While previous work summarized in Table 1 has extensively focused on pose estimation using LiDAR and stereo cameras, these systems, despite their accuracy, are often constrained by cost, weight, and complexity. Monocular vision offers a cost-effective and practical alternative but has not been thoroughly investigated regarding reliability and robustness. Although there has been substantial research in related areas, such as optical flow, depth estimation, and semantic segmentation, as outlined by the references, these studies have not adequately addressed the challenges of creating an adaptive, GPS-independent localization framework using monocular cameras. The gap, therefore, is in developing AI-driven monocular visual odometry solutions that achieve performance standards comparable to multi-sensor systems, offering a scalable, adaptable, and cost-effective solution across various platforms. This research aims to fill this gap by introducing a novel framework that leverages machine learning to enhance the capabilities of monocular vision systems for pose estimation, particularly in scenarios where additional sensors are not viable due to physical or financial constraints.

## 3. Vision-Base Pose Estimation

The architecture proposed in this paper leverages advanced computer vision techniques such as optical flow, depth estimation, and semantic segmentation, enhancing pose estimation accuracy. The proposed framework leverages these techniques to process image sequences from monocular cameras, addressing the challenges associated with dynamic environments and GPS-denied areas to estimate sensor pose changes from a sequence of frames captured by a single camera. In this section, we present the literature on integrating various computer vision techniques,

Zhi et al. [41] introduced an unsupervised monocular visual odometry method incorporating multiscale modeling to enhance pose and depth estimation accuracy, particularly in scenarios with varying motion patterns. The proposed framework utilizes densely linked atrous convolutions to expand the receptive field size while preserving image details. It integrates a nonlocal self-attention mechanism to capture long-range dependencies effectively. These architectural components enable the model to effectively model objects of different image scales, improving visual odometry accuracy, especially in rotating scenes. Despite the effectiveness of these components, their study highlights the inefficiency of rescaling for feature fusion in nonlocal modeling, suggesting a potential area for further optimization in future research. Liu et al. [42] proposed a Competition-Cooperation Transformer Network for the joint estimation of pose, depth, and optical flow, likely incorporating a transformer-based architecture. This architecture is designed to capture complex relationships between the visual perception tasks by integrating competitive and cooperative mechanisms. The network structure may consist of multiple transformer layers with attention mechanisms that enable the model to effectively process spatial and temporal information. This facilitates accurate pose, depth, and optical flow estimation in a unified framework. In the study by Zhao et al. [43] on Deep Direct Visual Odometry, the architecture likely involves a deep learning framework tailored for direct camera motion estimation from consecutive image frames. This architecture may consist of convolutional neural networks (CNNs) or recurrent neural networks (RNNs) designed to process visual data efficiently and accurately predict camera poses. The model may incorporate multiple layers with varying complexities to capture intricate spatial and temporal dependencies in the input data, enabling real-time and precise pose estimation for applications such as autonomous driving and robotics. In Song et al. [44], the ContextAVO method introduces a sophisticated architecture that leverages local context guidance to refine poses in deep visual odometry systems. The architecture likely incorporates convolutional neural networks (CNNs) or recurrent neural networks (RNNs) to process sequential image data and extract relevant features for pose estimation. Additionally, the model may include attention mechanisms to focus on critical regions in the input data, enhancing the accuracy and robustness of pose refinement in challenging visual odometry scenarios. Tu et al. [45] proposed EMA-VIO, a deep visual-inertial odometry system that integrates external memory attention mechanisms. The architecture likely consists of a deep neural network that fuses visual and inertial sensor data with external memory modules to capture long-term dependencies. The attention mechanism in EMA-VIO enables the model to focus on relevant information for accurate motion estimation, making it robust in challenging environments. In Xu et al. [46], the AirVO method is introduced as an illumination robust point-line visual odometry system with a unique architecture. AirVO combines point and line features in a carefully designed neural network structure to estimate camera motion accurately, particularly in challenging lighting conditions. The architecture likely includes modules for feature extraction, point-line correspondence establishment, and motion estimation, showcasing a sophisticated design tailored to handle varying illumination levels effectively. Additionally, the network may incorporate attention mechanisms or memory units to capture long-term dependencies and enhance the robustness of odometry estimation in diverse lighting environments. Zhu et al. [47] proposed DeepAVO, which refines poses in deep Visual Odometry through feature distillation. The architecture likely involves a deep neural network that processes visual data to estimate poses, focusing on distilling informative features to enhance accuracy. The network may consist of convolutional layers for feature extraction, followed by modules for feature distillation, enabling the model to refine pose estimates efficiently. Additionally, the architecture may incorporate attention mechanisms or recurrent layers to capture temporal dependencies in the visual data, contributing to the robustness and precision of pose estimation in challenging scenarios. Yang et al. [48] introduced the D3VO framework, which leverages deep networks for monocular visual odometry by incorporating deep depth, pose, and uncertainty estimation. The proposed self-supervised monocular depth estimation network enhances accuracy by aligning training image pairs under similar lighting conditions and modeling photometric uncertainties. This framework performs better than traditional methods and achieves results comparable to stereo/LiDAR odometry and visual-inertial odometry while utilizing only a single camera.

The literature summarized in Table 2 highlights several limitations in existing vision-based pose estimation methods that underscore the need for further advancements. Key challenges identified include inefficiencies in feature fusion (as noted in Zhi et al.’s unsupervised monocular visual odometry), high computational complexity (Liu et al.’s transformer-based joint estimation), and limited robustness in dynamic environments (Zhao et al.’s Deep Direct Visual Odometry). Additionally, approaches like EMA-VIO (Tu et al.) depend on inertial sensors, adding complexity to the system, while others, such as DeepAVO (Zhu et al.), are sensitive to visual noise and lighting inconsistencies. The reviewed methods generally excel in specific contexts but struggle to provide a cost-effective, scalable, and adaptable outdoor monocular pose estimation solution. These limitations lead to the development of the proposed pipeline in this paper, which seeks to address the gaps by introducing a robust, AI-driven monocular visual odometry system. This system integrates optical flow, depth estimation, and semantic segmentation to enhance performance in GPS-denied environments without additional sensors, making it scalable and adaptable across different platforms.

## 4. The Proposed Monocular Pose Estimator

This section introduces a novel pose estimation architecture designed explicitly for monocular camera setups. This architecture leverages advanced computer vision techniques and transformer models to estimate sensor pose changes from a sequence of frames captured by a single camera. The system is structured into distinct yet interconnected components, each optimized for handling specific tasks related to optical flow, depth estimation, semantic segmentation, and dynamic object filtering. These components work synergistically to accurately estimate the camera’s 6 degrees of freedom (6DoF) pose. The proposed system is built with robustness and efficiency by employing deep learning models for complex spatial and temporal feature extraction. Throughout this section, we will explain the architecture in detail, outlining the role of each block in the pipeline, their contributions to the overall system, and the benefits of combining these models for pose estimation. In the following subsections, we summarize the architecture’s key components, their functionalities, and how they interact to produce high-precision pose estimates. We will also highlight the advantages of this architecture, such as its ability to integrate depth, motion, and semantic data, as well as its capacity to adapt to various dynamic environments. 

### 4.1. Architecture

**Figure 1:**
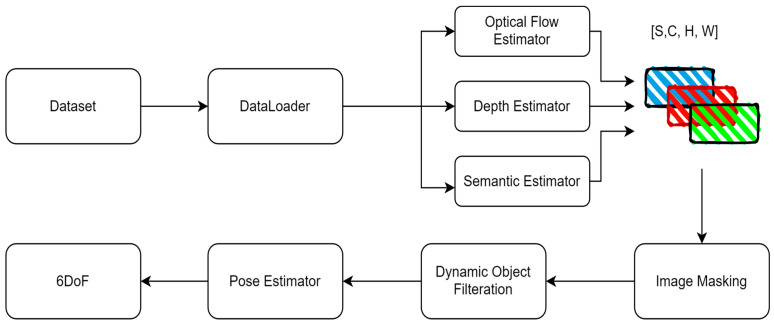
Proposed Pipeline Architecture.

Figure 1 describes the proposed pose estimation system that leverages advanced computer vision techniques and transformer models to estimate sensor pose changes from a sequence of frames captured by a monocular camera. Each block in the pipeline serves a specialized function, utilizing deep learning models optimized for their tasks. Following, we describe each block and its contributions to the system, as well as the advantages of this architecture.

**Figure 2:**
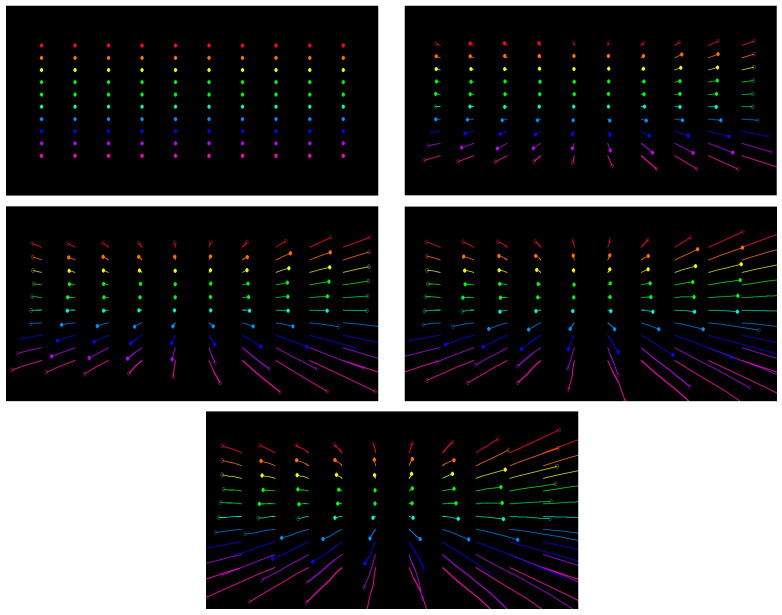
Optical flow processed output sample for one sequence of frames.

**Figure 3:**
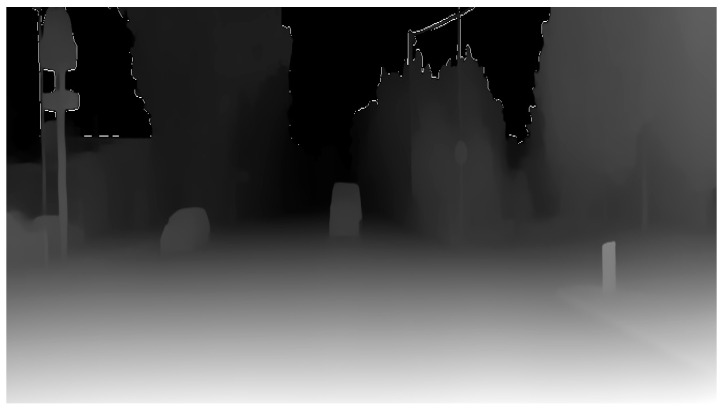
Sample output of depth estimation.

**Figure 4:**
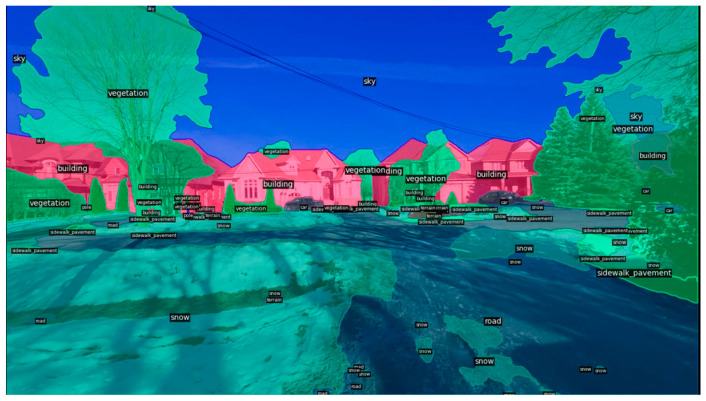
Sample output of the semantic segmentation.

**Figure 5:**
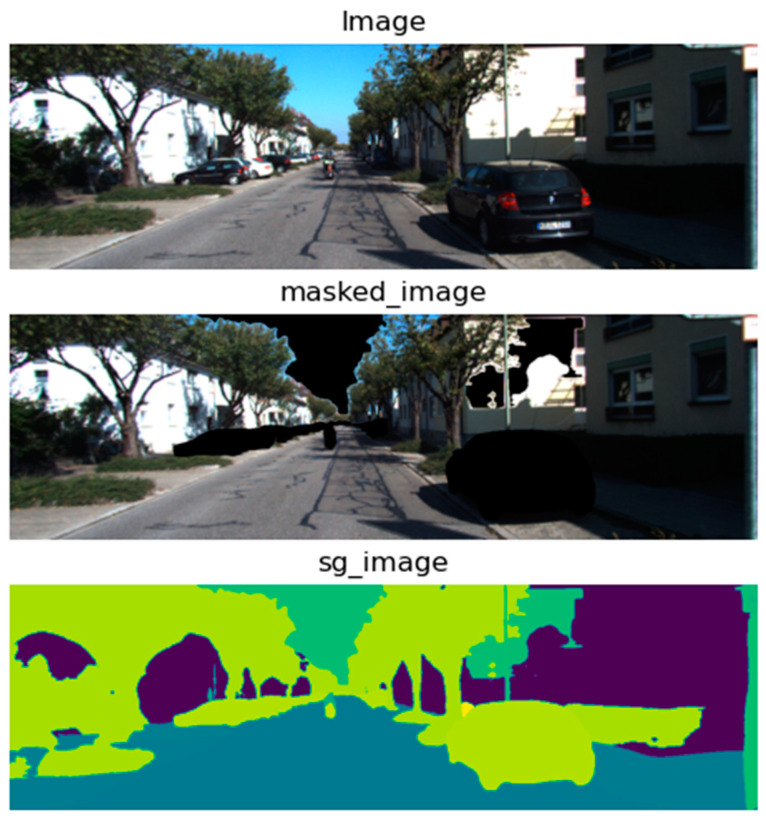
Sample output of dynamic object and sky removal.

- **Data Loader**: This component retrieves data from the dataset in manageable sizes for processing (denoted by n batches of pair images). It ensures efficient data handling during the training or inference stages.
- **Optical Flow Estimator**: CoTracker [49] is presumably a transformer-based model designed for tracking objects across frames by estimating their motion (optical flow). It provides a dense field of motion vectors that represent the displacement of pixels between consecutive frames. The estimator’s output goes to a ten-by-ten grid matrix for every five frames. The track of the motion is drawn on each frame. Finally, the original image on the frame is removed to reduce the processing for the pose estimator, as shown in Figure 2.

- **Depth Estimator** [50]: This block estimates the depth from the camera to each point in the image. The cited approach suggests an integrated model that combines information from optical flow and direct depth estimation to produce a depth map; a sample output of the depth estimation process is shown in Figure 3.

- **Semantic Estimator**: HRNet [32,51], which stands for High-Resolution Network, is a neural network architecture for semantic segmentation that maintains high-resolution representations throughout the network. This allows it to predict fine-grained semantic labels, which is crucial for understanding different objects and regions within the frame; a sample output of the semantic segmentation process is shown in Figure 4.

- **Masking**: Here, based on the information from optical flow, depth, and semantic estimations, a masking operation focuses on certain regions or objects of interest by filtering out irrelevant parts of the image.
- **Dynamic Object Filtration**: This step filters out dynamic objects, which could be crucial for focusing the pose estimation on the static background or separating moving objects’ influence from the camera’s motion. The categories of objects to be removed include sky, car, pedestrian, bicycle, and motorcycle, as shown in Figure 5.

- **Image Stacking**: This block likely stacks or aligns multiple frames to comprehensively represent the scene, considering changes in view due to camera and object movements.

**Figure 6:**
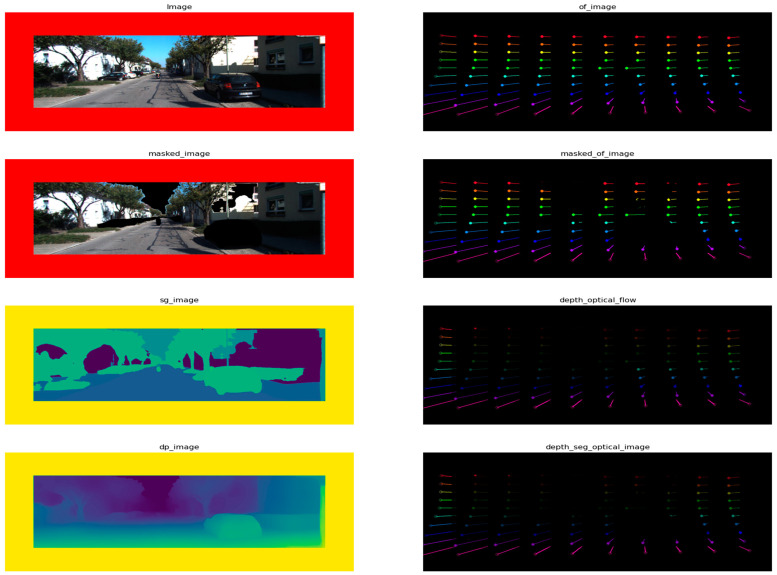
Step-by-step preprocessing samples.

Figure 6 shows the full step-by-step preprocess and the sample outcome. It shows the original image, the image with the removed sky, cars and bicycle, semantic map, depth map, optical flow movement tracks, optical flow masked with a semantic map, weighted optical flow with a depth map, and finally, the result of the whole preprocessing the optical flow with masked semantic and depth.

**Figure 7:**
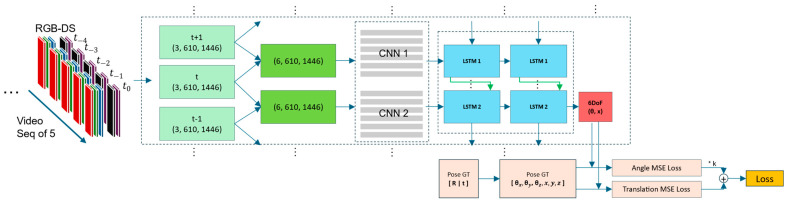
Pose estimator architecture. I changed it and replaced the image.

- **Pose Estimator:** The pose estimator network shown in Figure 7 processes a rich set of visual data to estimate a camera’s 6 degrees of freedom (6DoF) pose. The input to the network consists of an RGB optical flow image, a semantic segmentation map, and a depth map. These inputs are combined to comprehensively represent the scene across multiple frames. Initially, the input data is segmented into a sequence of frames, each representing five channels: three for the RGB optical flow, one for the semantic segmentation, and one for the depth map. These frames are then stacked in pairs to create a sequence of combined frames for each time step, labeled as t − 1, t, and t + 1, each with dimensions of (5, 610, 1446). The pairs of frames (t − 1, t) and (t, t + 1) are then processed to create stacked images with dimensions of (10, 610, 1446), which are fed into Convolutional Neural Networks (CNNs) to extract spatial features. The CNNs process these stacked frames to produce feature maps that capture the spatial structure of the scene. These feature maps are then passed to Long Short-Term Memory (LSTM) networks, which are specialized types of Recurrent Neural Networks (RNNs). The LSTM networks are designed to capture temporal dependencies and dynamics within the sequence of frames. Two layers of LSTMs, denoted as LSTM 1 and LSTM 2, are used to process the feature maps sequentially, capturing the evolution of the scene over time. The output from the LSTM layers consists of 6DoF pose estimates for each time step, represented as vectors of rotational and translational components (θ, x). These estimates are then compared to ground truth poses, which include both rotational matrices [R|t] for 12-DoF and individual rotational and translational components (θx, θy, θz, x, y, z) for 6-DoF. The network computes the Mean Squared Error (MSE) loss for the angle and translation estimates. These losses are combined and scaled by a constant factor to compute the final loss. The network is trained to minimize this loss, improving its accuracy in estimating the 6DoF poses from the given visual input data. This architecture effectively combines spatial and temporal information to provide robust pose estimations in dynamic environments.

### 4.2. Output Format

The final output provides the estimated pose changes, including translation (∆x, ∆y,∆z), rotation Roll (∆φ), Pitch (∆θ), Yaw (∆ψ), and velocity (∆v), which collectively describe the sensor’s movement.

### 4.3. Advantages of the Architecture

- **High Precision**: Transformers across the pipeline allow for precise motion, depth, and semantics estimations due to their ability to capture long-range dependencies and contextual information within data.
- **Integrated Depth and Motion Estimation**: By unifying flow, stereo, and depth estimation, the system likely benefits from a more cohesive and robust depth map informed by motion across frames.
- **Effective Semantic Segmentation**: HRNet’s ability to maintain high-resolution information through the network provides detailed and accurate semantic segmentation, which can significantly improve the quality of masking and dynamic object filtration.
- **Dynamic Object Handling**: Including a dedicated block for dynamic object filtration helps the system isolate the camera’s motion from the motion of objects in the environment, which is critical for accurate pose estimation.
- **Temporal Understanding**: Stacking images over time enables the model to understand the temporal dynamics of the scene better, leading to more accurate pose estimations.
- **Versatility**: The architecture can be adapted to various environments by retraining with suitable datasets.

The proposed architecture reflects a comprehensive approach to pose estimation, integrating various sophisticated computer vision techniques and the powerful capabilities of transformer models. It is designed to provide accurate and detailed pose estimations that could be used in various applications, from autonomous vehicles to augmented reality systems.

## 5. Experimental Results and Analysis

In this section, we present the results of our experiments and analyze the performance of the proposed adaptive framework for outdoor pose estimation. The experiments were conducted using the KITTI odometry dataset, which is widely recognized for its application in tasks like autonomous driving and visual odometry. We evaluated our framework using various preprocessing methods and hyperparameters, aiming to identify the most effective configuration for accurate pose estimation. Our results are detailed through comparisons of training and validation losses, sensitivity analyses, and evaluations of different learning rates, providing a comprehensive understanding of the model’s behavior and its performance across diverse conditions.

### 5.1. Experimental Setup

We conducted extensive experiments using the KITTI odometry dataset to evaluate the effectiveness of our proposed adaptive framework for outdoor pose estimation. These experiments were performed on an NVIDIA RTX 3090 GPU (Toronto, ON, Canada), ensuring efficient training and testing processes. The training was carried out over 200 epochs with a learning rate of 0.008, typically requiring 9 to 14 h per training session. The Adagrad optimizer was employed alongside the ReLU activation function to optimize the model parameters.

### 5.2. Dataset Division

The KITTI odometry dataset is a widely used benchmark in computer vision and robotics, particularly for tasks involving autonomous driving and visual odometry. This dataset provides raw data from a moving vehicle, including stereo camera images, 3D laser scans, and GPS/IMU data. A crucial aspect of visual odometry involves estimating the vehicle’s motion, represented by the transformation matrix [R|t], where *R* is a 3 × 3 rotation matrix, and *t* is a 3 × 1 translation vector. The [R|t] matrix is used to transform a point *p* in the coordinate frame of one camera position to another camera position as follows:

(1) $$\overset{´}{p=}\;Rp+t$$

Here, *p* is the point in the original coordinate frame, *R* rotates this point, and *t* translates it. For example, given a sequence of images from the KITTI dataset, the visual odometry algorithm aims to estimate the successive [R|t] matrices that describe the vehicle’s trajectory through the environment. Accurate estimation of these matrices is essential for applications such as 3D mapping, navigation, and autonomous driving, providing a basis for understanding and interpreting the vehicle’s movement in real-world scenarios. The KITTI odometry dataset was divided into training, validation, and testing subsets to facilitate a robust evaluation of our models. Specifically, the training subset consisted of sequences [00, 01, 02, 05, 08, 09], which are relatively long and provide substantial data for learning robust features. For validation, sequences [04, 06, 07, 10] were used to tune the model and prevent overfitting. Finally, all available sequences were used for testing to comprehensively evaluate our model’s performance.

### 5.3. Preprocessing Methods

We explored four different preprocessing methods to assess their impact on pose estimation accuracy.

1. **Base Model (Brown Color)**: This baseline model does not utilize preprocessing steps and directly inputs raw images into the neural network. It is the original architecture of DeepVO [52].
2. **Optical Flow Only (Yellow Color)**: This method involves computing optical flow between consecutive frames to capture motion information, which is then used as input to the model.
3. **Optical Flow with Depth Estimation (Green Color)**: In this approach, optical flow and depth maps are computed for each frame pair. The depth estimation provides additional spatial information about the scene, improving the model’s understanding of the environment.
4. **Optical Flow with Depth and Semantic Segmentation (Purple Color)**: This comprehensive preprocessing method combines optical flow, depth estimation, and semantic segmentation. The semantic segmentation provides context about different objects in the scene, further enhancing the model’s capability to interpret complex environments.

### 5.4. Evaluation Metrics

The performance of each preprocessing method was evaluated using the mean squared error (MSE) for both translation and rotation. The combined error metric used in our analysis is:

(2) Error=Translation MSE+k×Rotation MSE

where *k* = 100 to appropriately scale the rotation error for aggregation with the translation error.

### 5.5. Training Results

**Figure 8:**
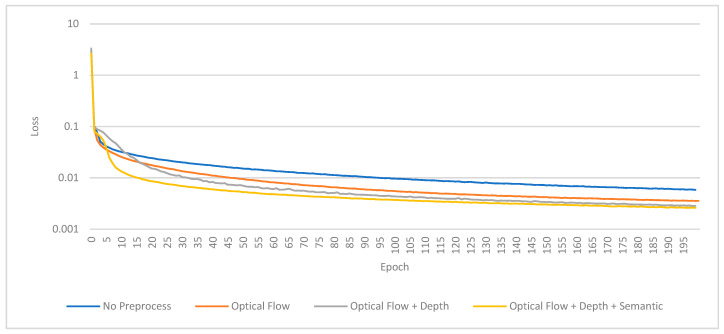
Train/Loss chart for the KITTI odometry dataset.

**Figure 9:**
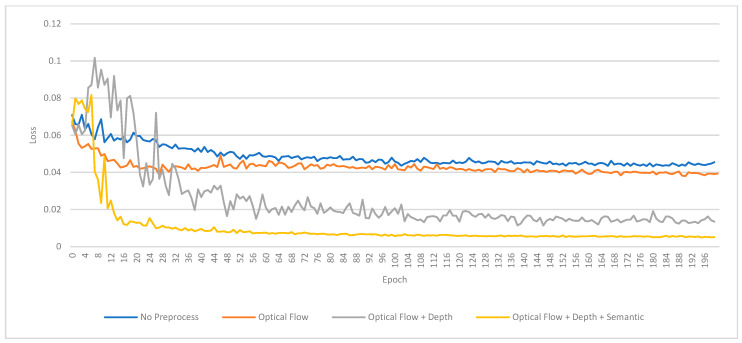
The validation/Loss chart for the KITTI odometry dataset shows that the pose estimator can learn more rapidly by providing extra scene information, especially semantic segmentation, to add correction weight to each class of objects and remove dynamic ones from the estimations.

**Figure 10:**
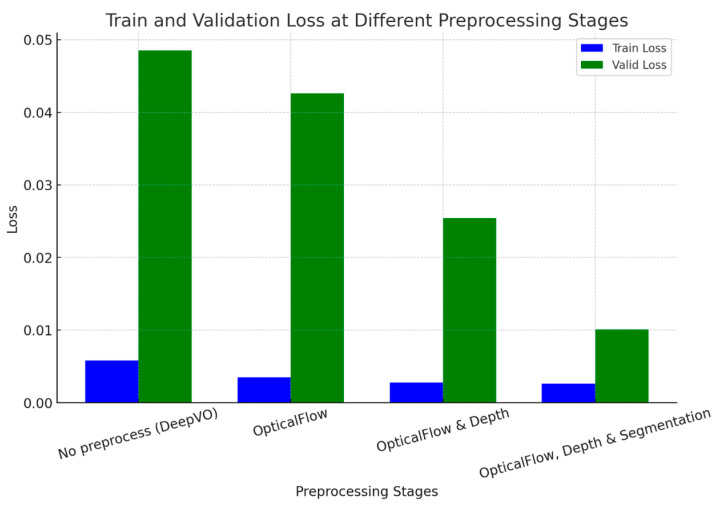
Train and validation loss for different preprocessing stages, including no preprocessing, OF, OF with depth estimation, and OF with depth and semantic segmentation.

**Figure 11:**
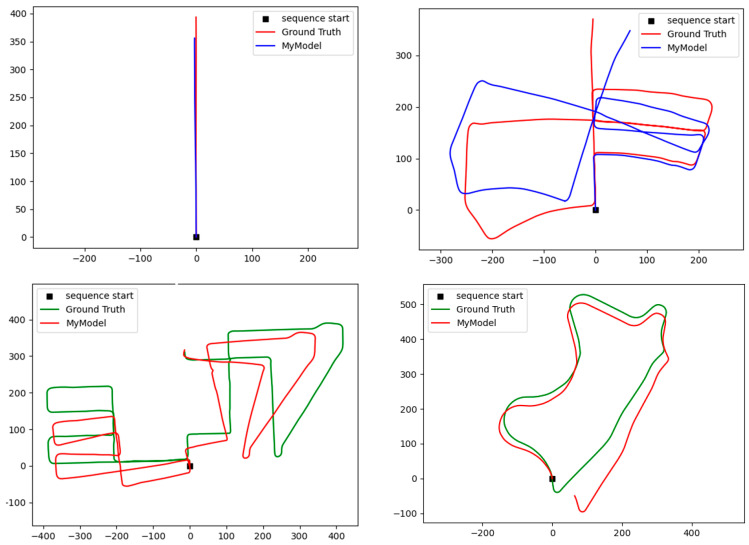
Proposed model’s tracking experience output for the KITTI odometry dataset. The *X* and *Y*-axis units are in meters.

The results of our experiments are summarized in Figure 8, Figure 9, Figure 10 and Figure 11 and Table 3, which depict the training and validation loss for each preprocessing method. In the training loss chart Figure 8, the base model (brown) demonstrates the highest loss throughout the epochs, indicating its inferior performance without preprocessing. The optical flow-only model (yellow) significantly improves over the base model. Further enhancements are observed with the optical flow combined with depth estimation (green). The model achieves the best performance using optical flow, depth, and semantic segmentation (purple), which consistently exhibits the lowest loss values. The validation loss chart in Figure 9 corroborates these findings. The base model again performs the worst, while the optical flow-only model performs better but shows some fluctuation in the early epochs. The optical flow and depth estimation model achieves a more stable and lower validation loss. The model integrating optical flow, depth, and semantic segmentation continues to outperform the others, maintaining the lowest validation loss across the epochs. These results indicate that incorporating advanced preprocessing techniques, particularly optical flow, depth estimation, and semantic segmentation, substantially improves the pose estimation performance. This validates the effectiveness of our proposed framework and its potential for robust outdoor pose estimation.

Table 3 presents the loss values of our visual odometry model across different preprocessing techniques, demonstrating a clear performance hierarchy. Without preprocessing, the baseline model (DeepVO) exhibits the highest losses across almost all sequences, with a training loss of 0.0058 and a validation loss of 0.0485. Introducing optical flow as a preprocessing step significantly reduces the losses, achieving a training loss of 0.0035 and a validation loss of 0.0426. Further enhancement is observed when depth estimation is incorporated alongside optical flow, lowering the training and validation losses to 0.0028 and 0.0254, respectively. The most substantial improvement is seen with semantic segmentation in addition to optical flow and depth estimation, resulting in the lowest training loss of 0.0026 and a validation loss of 0.0101. This comprehensive preprocessing approach consistently yields the lowest losses across all sequences, underscoring its effectiveness in enhancing model performance for visual odometry tasks.

Figure 9 shows the validation/loss chart for the KITTI odometry dataset, which shows that the pose estimator can learn more rapidly by providing extra scene information, especially semantic segmentation, to add correction weight to each class of objects and remove dynamic ones from the estimations.

Figure 10 presents a bar graph showing the train and validation loss for different preprocessing stages, including no preprocessing, OF, OF with depth estimation, and OF with depth and semantic segmentation. It shows adding extra information to the pose estimator helps it to have a more accurate 6DoF estimation. It also significantly lowers the enormous gap between training and validation loss. This means that preprocessing helps generalization considerably. 

Figure 11 provides the proposed model’s tracking experience output for the KITTI odometry dataset. The *X* and *Y*-axis units are in meters. The drifting error appears after a few hundred meters. It is the main challenge in visual odometry. As presented, the translation estimation is accurate, but the rotation estimation still needs to be more accurate to map the trajectories precisely.

### 5.6. Sensitivity Analysis

In this analysis, we explore the impact of different learning rates on the training and validation performance of a 6DoF pose estimation model. The learning rates examined are 0.008, 0.005, 0.011, and 0.014. The objective is to determine the optimal learning rate that balances rapid convergence and stable performance across training and validation datasets.

#### 5.6.1. Training Loss Analysis

**Figure 12:**
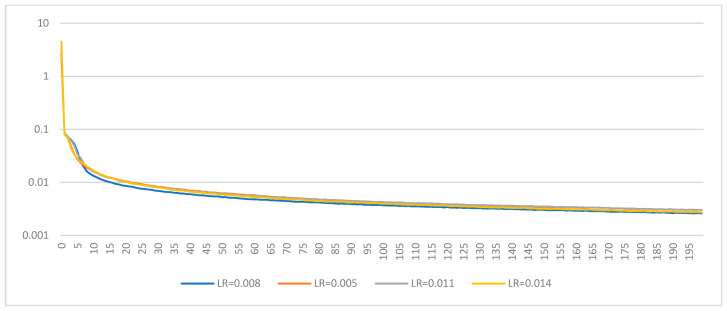
Train/Loss with different learning rates.

The training loss curves for the different learning rates are displayed in Figure 12. Observing the curves provides insight into how each learning rate affects the model’s ability to minimize loss during training.

(1) **LR: 0.008**: The training loss starts high and decreases steadily, indicating a good convergence rate. The curve shows a smooth decline, suggesting a stable training process.
(2) **LR: 0.005**: This learning rate also demonstrates a steady decrease in training loss. The curve is similar to LR: 0.008 but with a slightly slower convergence rate due to the lower learning rate.
(3) **LR: 0.011**: The training loss for this learning rate decreases rapidly initially but shows more fluctuations compared to the previous two. This suggests that the higher learning rate introduces instability while the convergence is fast.
(4) **LR: 0.014**: The highest learning rate among the four shows the most rapid initial decrease in training loss. However, this curve also displays more pronounced fluctuations, indicating potential instability during training.

#### 5.6.2. Validation Loss Analysis

**Figure 13:**
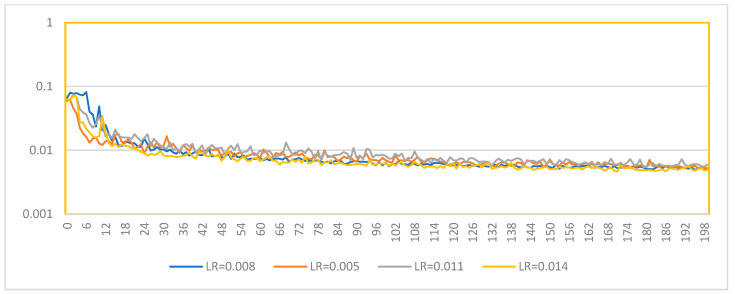
Validation/Loss with different learning rates.

The validation loss curves indicate how well the model generalizes to unseen data. These curves are shown in Figure 13.

(1) **LR: 0.008**: The validation loss decreases steadily but exhibits some fluctuations. This indicates a good generalization with some overfitting tendencies, which might be addressed with further regularization techniques.
(2) **LR: 0.005**: The validation loss shows a smooth decline with fewer fluctuations than LR: 0.008, suggesting a better generalization capability with reduced overfitting.
(3) **LR: 0.011**: Similar to the training loss, the validation loss for this learning rate decreases rapidly but with noticeable fluctuations. This highlights the tradeoff between fast convergence and stability.
(4) **LR: 0.014**: The validation loss curve shows the most significant fluctuations, indicating that while the model converges quickly, it may suffer from substantial overfitting and instability issues.

Our analysis reveals that lower learning rates (0.005 and 0.008) offer a more stable training and validation process with less fluctuation in the loss curves. Among these, LR: 0.005, with a learning rate of 0.005, appears to provide the best balance between convergence speed and stability, showing smoother curves with fewer fluctuations. Higher learning rates (0.011 and 0.014) enable faster convergence but at the cost of increased instability and potential overfitting, as indicated by the more pronounced fluctuations in training and validation loss curves. Therefore, a learning rate of 0.005 is recommended for the 6DoF pose estimation task. This rate provides a good balance, ensuring stable and generalizable performance.

## 6. Conclusions

In conclusion, our experiments on the KITTI odometry dataset demonstrate the benefits of incorporating sophisticated preprocessing methods into the pose estimation pipeline. Our results highlight the importance of leveraging multiple sources of information, such as optical flow, depth, and semantic segmentation, to achieve accurate and reliable pose estimation in challenging outdoor environments. Moreover, a substantial body of research and numerous papers focused on pose estimation using LiDAR or stereo cameras, which leverage multiple viewpoints or sensor types to enhance accuracy and robustness. However, few studies address pose estimation using mono images, where only a single camera is employed. This gap is significant because many drones, cars, and other vehicles are equipped with just one camera, making it essential to develop models that can deliver reliable and accurate pose estimation from this limited data source. Our work aims to fill this gap by proposing a novel approach for pose estimation using mono images. Focusing on a single-camera setup addresses a critical need in the field, ensuring that our model can be widely applicable to various platforms and systems that rely on a single visual input. This contribution is significant for cost-effective and resource-constrained applications where deploying additional sensors may not be feasible.

## Figures and Tables

**Table 1:** A Comparative Summary of Existing Methods.

Category	Ref.	Method	Key Features
Optical Flow	[1]	Graph reasoning for motion estimation	Decouples context reasoning from matching
	[2]	Consistency on distrained pairs	Addresses distrained pairs
	[3]	Deep equilibrium models	Equilibrium via dynamic systems
	[4]	Transformer with MCVA	Cost volume autoencoding
	[5]	Event camera data	Event camera for fast motion
	[6]	Motion disentangling	Disentangling motion components
	[7]	Kernel Patch Attention	Focus on informative regions
	[8]	Global Matching with Overlapping Attention	Overlapping attention for global matching
	[9]	Spiking cameras	Uses spiking camera data
	[10]	Survey on video super-resolution	Optical flow for video enhancement
	[11]	Unsupervised learning	Unsupervised learning for optical flow
	[12]	Multiplane images	Leverages multiplane images
	[13]	EM for dataset generation	EM-based dataset generation
	[14]	Gaussian attention	Incorporates Gaussian attention
	[15]	Global Matching via softmax	Global matching with softmax
	[16]	VideoFlow with temporal info	Temporal coherence
	[17]	Geometric matching	Geometric constraints in matching
	[18]	Deep Inverse PatchMatch	Patchmatch refinement
	[19]	Cross-Attentional Flow Transformer	Cross-attention for robust flow
Depth Estimation	[20]	Self-supervised monocular depth	Self-supervised depth estimation
	[21]	Explicit depth supervision	Improves accuracy with depth supervision
	[22]	Transformer-CNN hybrid	Combines Transformer with CNN
	[23]	Adaptive bins	Adaptive depth bins
	[24]	Self-supervised with depth cues	Depth cues for segmentation
	[25]	Wavelet decomposition	Wavelet-based depth prediction
	[26]	CRFs with contextual refinement	CRF for depth refinement
	[27]	Transformer for stereo	It uses a transformer for stereo-depth
	[28]	Self-supervised vision transformer	Vision transformer for depth
	[29]	Attention-based aggregation	Attention to contextual aggregation
	[30]	Content adaptive multi-res merging	Merging for high-res depth
	[31]	Self-supervised depth estimation	Self-supervised depth for segmentation
Semantic Segmentation	[32]	High-resolution representation learning	High-res representations
	[33]	Real-time BiSeNet reevaluation	Optimized for real-time performance
	[34]	Progressive semantic segmentation	Progressive refinement
	[35]	PID controller-based network	PID control for segmentation
	[36]	HRNet and PSPNet for multiband	Multiband segmentation with HRNet
	[37]	Transformer for segmentation	Transformer-based model
	[38]	Object context for segmentation	Object context integration
	[39]	Optimized HRNet	Optimized HRNet for segmentation
	[40]	High-resolution with dual attention	High-res and boundary-aware

**Table 2:** Comparing Vision-based VO methods.

Study	Method	Key Features	Strengths	Limitations
[41]	Unsupervised Monocular Visual Odometry	Multiscale modeling with atrous convolutions, nonlocal self-attention	Enhances pose and depth estimation in rotating scenes	Inefficiencies in rescaling for feature fusion
[42]	Competition-Cooperation Transformer Network	Transformer-based architecture for the joint estimation of pose, depth, and optical flow	Captures complex visual perception tasks	High computational complexity
[43]	Deep Direct Visual Odometry	CNN/RNN-based deep learning framework for direct camera motion estimation	Real-time pose estimation for autonomous driving	Limited robustness in dynamic environments
[44]	ContextAVO	Local context guidance for pose refinement, CNN/RNN-based architecture	Refines poses in visual odometry with attention	Performance may degrade in highly dynamic scenarios
[45]	EMA-VIO	Deep visual-inertial odometry with external memory attention	Robust to long-term dependencies, sensor fusion	Requires inertial sensors, increasing system complexity
[46]	AirVO	Point-line feature combination for illumination robust odometry	Effective in varying illumination conditions	Sensitive to feature quality, may underperform in low-texture areas
[47]	DeepAVO	Deep learning-based pose refinement with feature distillation	Efficient pose refinement	Sensitive to visual noise and inconsistent lighting
[48]	D3VO	Deep depth, pose, and uncertainty estimation, self-supervised	High accuracy comparable to stereo/LiDAR systems	Limited adaptability across different cameras and environments

**Table 3:** Test results on the KITTI dataset.

Preprocessing ^1^	Seq 00	Seq 01	Seq 02	Seq 04	Seq 05	Seq 06	Seq 07	Seq 08	Seq 09	Seq 10
No preprocess (DeepVO)	45.58	257.7	625.7	37.33	70.95	231.5	80.48	91.39	177.5	577.1
OpticalFlow	3.806	56.50	72.57	252.9	13.56	305.4	15.22	24.79	72.17	1369
OpticalFlow & Depth	13.41	305.2	159.6	315.6	64.52	95.43	3.799	58.87	41.62	246.2
OpticalFlow, Depth & Segmentation	3.767	28.09	9.695	10.48	16.20	4.312	6.573	0.643	30.43	30.00

^1^ In the KITTI dataset, sequences 00 to 10 are part of the odometry benchmark and consist of stereo image data, LiDAR scans, and ground truth poses captured during urban and suburban driving scenarios. These sequences provide diverse challenges, including varying road types, lighting conditions, and dynamic objects.

## Data Availability

Data are contained within the article.

## References

[1] Luo A., Yang F., Luo K., Li X., Fan H., Liu S. (2022). Learning Optical Flow with Adaptive Graph Reasoning. Proc. AAAI Conf. Artif. Intell..

[2] Jeong J., Lin J.M., Porikli F., Kwak N. Imposing Consistency for Optical Flow Estimation. Proceedings of the IEEE Computer Society Conference on Computer Vision and Pattern Recognition.

[3] Bai S., Geng Z., Savani Y., Kolter J.Z. Deep Equilibrium Optical Flow Estimation. Proceedings of the 2022 IEEE Computer Society Conference on Computer Vision and Pattern Recognition.

[4] Huang Z., Shi X., Zhang C., Wang Q., Cheung K.C., Qin H., Li H. (2022). FlowFormer: A Transformer Architecture for Optical Flow.

[5] Wan Z., Dai Y., Mao Y. (2022). Learning Dense and Continuous Optical Flow From an Event Camera. IEEE Trans. Image Process..

[6] Deng C., Luo A., Huang H., Ma S., Liu J., Liu S. Explicit Motion Disentangling for Efficient Optical Flow Estimation. Proceedings of the 2023 IEEE International Conference on Computer Vision.

[7] Luo A., Yang F., Li X., Liu S. Learning Optical Flow with Kernel Patch Attention. Proceedings of the 2022 IEEE Computer Society Conference on Computer Vision and Pattern Recognition.

[8] Zhao S., Zhao L., Zhang Z., Zhou E., Metaxas D. Global Matching with Overlapping Attention for Optical Flow Estimation. Proceedings of the 2022 the IEEE Computer Society Conference on Computer Vision and Pattern Recognition.

[9] Hu L., Zhao R., Ding Z., Ma L., Shi B., Xiong R., Huang T. Optical Flow Estimation for Spiking Camera. Proceedings of the 2022 IEEE Computer Society Conference on Computer Vision and Pattern Recognition.

[10] Tu Z., Li H., Xie W., Liu Y., Zhang S., Li B., Yuan J. (2022). Optical flow for video super-resolution: A survey. Artif. Intell. Rev..

[11] Guizilini V., Lee K.-H., Ambrus R., Gaidon A. (2022). Learning Optical Flow, Depth, and Scene Flow Without Real-World Labels. IEEE Robot. Autom. Lett..

[12] Liang Y., Liu J., Zhang D., Fu Y. MPI-Flow: Learning Realistic Optical Flow with Multiplane Images. Proceedings of the 2023 IEEE International Conference on Computer Vision.

[13] Han Y., Luo K., Luo A., Liu J., Fan H., Luo G., Liu S. (2022). RealFlow: EM-Based Realistic Optical Flow Dataset Generation from Videos.

[14] Luo A., Yang F., Li X., Nie L., Lin C., Fan H., Liu S. GAFlow: Incorporating Gaussian Attention into Optical Flow. Proceedings of the 2023 IEEE International Conference on Computer Vision.

[15] Xu H., Zhang J., Cai J., Rezatofighi H., Tao D. GMFlow: Learning Optical Flow via Global Matching. Proceedings of the 2022 IEEE Computer Society Conference on Computer Vision and Pattern Recognition.

[16] Shi X., Huang Z., Li D., Zhang M., Cheung K.C., See S., Qin H., Dai J., Li H. (2023). FlowFormer++: Masked Cost Volume Autoencoding for Pretraining Optical Flow Estimation. arXiv.

[17] Dong Q., Cao C., Fu Y. Rethinking Optical Flow from Geometric Matching Consistent Perspective. Proceedings of the 2023 IEEE Computer Society Conference on Computer Vision and Pattern Recognition.

[18] Zheng Z., Nie N., Ling Z., Xiong P., Liu J., Wang H., Li J. DIP: Deep Inverse Patchmatch for High-Resolution Optical Flow. Proceedings of the 2022 IEEE Computer Society Conference on Computer Vision and Pattern Recognition.

[19] Sui X., Li S., Geng X., Wu Y., Xu X., Liu Y., Goh R., Zhu H. CRAFT: Cross-Attentional Flow Transformer for Robust Optical Flow. Proceedings of the 2022 IEEE Computer Society Conference on Computer Vision and Pattern Recognition.

[20] Lyu X., Liu L., Wang M., Kong X., Liu L., Liu Y., Chen X., Yuan Y. HR-Depth: High Resolution Self-Supervised Monocular Depth Estimation. Proceedings of the 35th AAAI Conference on Artificial Intelligence.

[21] Li Y., Ge Z., Yu G., Yang J., Wang Z., Shi Y., Sun J., Li Z. (2023). BEVDepth: Acquisition of Reliable Depth for Multi-View 3D Object Detection. Proc. AAAI Conf. Artif. Intell..

[22] Li Z., Chen Z., Liu X., Jiang J. (2023). DepthFormer: Exploiting Long-range Correlation and Local Information for Accurate Monocular Depth Estimation. Mach. Intell. Res..

[23] Bhat S.F., Alhashim I., Wonka P. AdaBins: Depth Estimation Using Adaptive Bins. Proceedings of the 2021 IEEE Computer Society Conference on Computer Vision and Pattern Recognition.

[24] Hoyer L., Dai D., Wang Q., Chen Y., Van Gool L. (2023). Improving Semi-Supervised and Domain-Adaptive Semantic Segmentation with Self-Supervised Depth Estimation. Int. J. Comput. Vis..

[25] Ramamonjisoa M., Firman M., Watson J., Lepetit V., Turmukhambetov D. Single Image Depth Prediction with Wavelet Decomposition. Proceedings of the 2021 IEEE Computer Society Conference on Computer Vision and Pattern Recognition.

[26] Yuan W., Gu X., Dai Z., Zhu S., Tan P. Neural Window Fully-connected CRFs for Monocular Depth Estimation. Proceedings of the 2022 IEEE Computer Society Conference on Computer Vision and Pattern Recognition.

[27] Li Z., Liu X., Drenkow N., Ding A., Creighton F.X., Taylor R.H., Unberath M. Revisiting Stereo Depth Estimation From a Sequence-to-Sequence Perspective with Transformers. Proceedings of the 2021 IEEE International Conference on Computer Vision.

[28] Zhao C., Zhang Y., Poggi M., Tosi F., Guo X., Zhu Z., Huang G., Tang Y., Mattoccia S. MonoViT: Self-Supervised Monocular Depth Estimation with a Vision Transformer. Proceedings of the 2022 International Conference on 3D Vision (3DV).

[29] Chen Y., Zhao H., Hu Z., Peng J. (2021). Attention-based context aggregation network for monocular depth estimation. Int. J. Mach. Learn. Cybern..

[30] Miangoleh S.M.H., Dille S., Mai L., Paris S., Aksoy Y. Boosting Monocular Depth Estimation Models to High-resolution via Content-adaptive Multi-Resolution Merging. Proceedings of the 2021 IEEE Computer Society Conference on Computer Vision and Pattern Recognition.

[31] Hoyer L., Dai D., Chen Y., Koring A., Saha S., Van Gool L. Three Ways to Improve Semantic Segmentation with Self-Supervised Depth Estimation. Proceedings of the 2021 IEEE Computer Society Conference on Computer Vision and Pattern Recognition.

[32] Wang J., Sun K., Cheng T., Jiang B., Deng C., Zhao Y., Liu D., Mu Y., Tan M., Wang X. (2021). Deep High-Resolution Representation Learning for Visual Recognition. IEEE Trans. Pattern Anal. Mach. Intell..

[33] Fan M., Lai S., Huang J., Wei X., Chai Z., Luo J., Wei X. Rethinking BiSeNet For Real-time Semantic Segmentation. Proceedings of the 2021 IEEE Computer Society Conference on Computer Vision and Pattern Recognition.

[34] Huynh C., Tran A.T., Luu K., Hoai M. Progressive Semantic Segmentation. Proceedings of the 2021 IEEE Computer Society Conference on Computer Vision and Pattern Recognition.

[35] Xu J., Xiong Z., Bhattacharyya S.P. PIDNet: A Real-time Semantic Segmentation Network Inspired by PID Controllers. Proceedings of the 2023 IEEE Computer Society Conference on Computer Vision and Pattern Recognition.

[36] Sun Y., Zheng W. (2023). HRNet- and PSPNet-based multiband semantic segmentation of remote sensing images. Neural Comput. Appl..

[37] Strudel R., Garcia R., Laptev I., Schmid C. Segmenter: Transformer for Semantic Segmentation. Proceedings of the 2021 IEEE International Conference on Computer Vision.

[38] Yuan Y., Huang L., Guo J., Zhang C., Chen X., Wang J. (2021). OCNet: Object Context for Semantic Segmentation. Int. J. Comput. Vis..

[39] Wu H., Liang C., Liu M., Wen Z. (2020). Optimized HRNet for image semantic segmentation. Expert Syst. Appl..

[40] Xu Z., Zhang W., Zhang T., Li J. (2021). HRCNet: High-resolution context extraction network for semantic segmentation of remote sensing images. Remote Sens..

[41] Zhi H., Yin C., Li H., Pang S. (2022). An Unsupervised Monocular Visual Odometry Based on Multi-Scale Modeling. Sensors.

[42] Liu X., Zhang T., Liu M. (2024). Joint estimation of pose, depth, and optical flow with a competition–cooperation transformer network. Neural Netw..

[43] Zhao C., Tang Y., Sun Q., Vasilakos A.V. (2022). Deep Direct Visual Odometry. IEEE Trans. Intell. Transp. Syst..

[44] Song R., Zhu R., Xiao Z., Yan B. (2023). ContextAVO: Local context guided and refining poses for deep visual odometry. Neurocomputing.

[45] Tu Z., Chen C., Pan X., Liu R., Cui J., Mao J. (2022). EMA-VIO: Deep Visual–Inertial Odometry With External Memory Attention. IEEE Sens. J..

[46] Xu K., Hao Y., Yuan S., Wang C., Xie L. AirVO: An Illumination-Robust Point-Line Visual Odometry. Proceedings of the 2023 IEEE International Conference on Intelligent Robots and Systems.

[47] Zhu R., Yang M., Liu W., Song R., Yan B., Xiao Z. (2022). DeepAVO: Efficient pose refining with feature distilling for deep Visual Odometry. Neurocomputing.

[48] Yang N., von Stumberg L., Wang R., Cremers D. D3VO: Deep Depth, Deep Pose and Deep Uncertainty for Monocular Visual Odometry. Proceedings of the 2020 IEEE Computer Society Conference on Computer Vision and Pattern Recognition.

[49] Karaev N., Rocco I., Graham B., Neverova N., Vedaldi A., Rupprecht C. (2023). CoTracker: It is Better to Track Together. arXiv.

[50] Xu H., Zhang J., Cai J., Rezatofighi H., Yu F., Tao D., Geiger A. (2023). Unifying Flow, Stereo and Depth Estimation. IEEE Trans. Pattern Anal. Mach. Intell..

[51] Lambert J.W., Liu Z., Sener O., Hays J., Koltun V. (2023). MSeg: A Composite Dataset for Multi-Domain Semantic Segmentation. IEEE Trans. Pattern Anal. Mach. Intell..

[52] Wang S., Clark R., Wen H., Trigoni N. (2017). Deepvo: Towards end-to-end visual odometry with deep Recurrent Convolutional Neural Networks. Proceedings of the 2017 IEEE International Conference on Robotics and Automation (ICRA).

